# Intra-arterial thrombolysis as an adjunct to thrombectomy in acute ischemic stroke: Encouraging but not conclusive evidence

**DOI:** 10.1093/esj/23969873251343814

**Published:** 2026-01-01

**Authors:** Adnan I Qureshi

**Affiliations:** Zeenat Qureshi Stroke Institute, Columbia, MO, USA; Department of Neurology, University of Missouri, Columbia, MO, USA

**Keywords:** Intra-arterial thrombolysis, thrombectomy, acute ischemic stroke, modified rankin scale, randomized controlled trials, symptomatic intracerebral hemorrhage%

Intra-arterial thrombolysis (IAT) has been used as an adjunct to mechanical thrombectomy (MT) in acute ischemic stroke patients for nearly two decades to improve distal arterial and microvascular perfusion, even in patients with near complete or complete recanalization, or enhance recanalization in patients with no or partial recanalization without any conclusive evidence.^1^ To provide the necessary evidence, 10 randomized controlled trials (RCTs) were initiated of which 8 were in China, 1 in Europe and 1 in Australia.^1^ Recent publications of several of these RCTs have provided an opportunity to combine the trials using meta-analysis to provide a more precise and robust estimate of the therapeutic effect of IAT. Yang et al.^2^ performed a meta-analysis of 4 RCTs, Chemical Optimization of Cerebral Embolectomy (CHOICE), Intra-Arterial Tenecteplase Following Endovascular Reperfusion for Large Vessel Occlusion Acute Ischemic Stroke (POST-TNK), Adjunctive Intra-Arterial Urokinase After Successful Endovascular Thrombectomy in Patients With Large-Vessel Occlusion Stroke (POST-UK), and Endovascular Recanalization in Patients with Acute Posterior Circulation Arterial Occlusion (ATTENTION-IA) involving 1395 patients. The analysis demonstrated that patients who were treated with IAT and MT had a higher likelihood of achieving an excellent functional outcome (defined by modified Rankin Scale [mRS] of 0–1) at 90 days compared to those who received MT alone. However, there was no difference in the rate of functional independence (defined by mRS 0–2) at 90 days in patients treated with IAT and MT compared with those treated with MT alone. The results should be interpreted with the understanding that the study population varied in clinical characteristics so the results cannot be generalized to a particular acute ischemic stroke patient population and formal adjustment for multiple comparisons was not performed in these analyses. The absolute increase in proportion of patients who achieved excellent functional outcomes was approximately 7% with IAT and MT (compared with MT alone). Furthermore, the benefit was seen only in the proportion of patients achieving excellent functional outcomes but not in other efficacy endpoints. The value of higher proportion of patients achieving mRS 0–1 requires additional consideration. The mRS score of 0–1 translates into 0.09 quality-adjusted-life-years (QALYs) gained in the first year and a mean 0.45 QALYs per subject over 5 years.^3^ The health-related quality of life perceived by patients is significantly better for those with mRS of 0–1 as opposed to those with other grades supporting the therapeutic value from patients’ perspective. From a safety perspective, the rate of symptomatic intracranial hemorrhage (sICH) was higher in the patients treated with IAT and MT (5.2%) than in the patients treated with MT alone (4.1%). The definition of sICH varied across the four studies and formal testing to determine if sample size was adequate to exclude non-inferiority regarding sICH rates was not performed. Interestingly, the benefit of IAT was greater in women than in men, a finding which remains unexplained in the absence of any clear pathophysiological explanation.

There is a broader question of whether the design of the RCTs evaluating the therapeutic benefit of IAT have resulted in meta-analysis as the only option to adequately address the question. The ongoing and completed RCTs have sample sizes ranging from 80 to 498 that can identify very large minimal clinically important differences (MCID; 13%–20% increase). Such sample sizes are inadequate to detect smaller MCIDs (<10%) that can still result in practice changes with low cost and complexity and easy-to-implement interventions such as IAT, leading to inconclusive results. The large non-inferiority margins due to small sample size means that the IAT can have substantially higher rates of sICH and still be considered “non-inferior” to MT alone. Therefore, the flaws in the design of existing RCTs result in missed opportunities which may not provide conclusive evidence regarding the efficacy and safety of IAT. Zarin et al.^4^ labeled these flaws as preventable un-informativeness in RCTs and urged caution regarding violating research ethics from patient’s perspective. Therefore, the only option to overcome the limitations requires combining data from ongoing or planned RCTs 3–5 years from now using aggregate level meta-analysis, network meta-analysis, or pooled analysis of patient-level data assuming that these RCTs are relatively homogenous in their design and selection criteria with limited publication biases. Network meta-analyses require high level of network consistency (consistent results from direct and indirect comparisons). Pooled analysis of patient-level data requires data elements to be shared and harmonized (common definitions) between RCTs.

The meta-analysis included RCTs which are randomizing acute ischemic stroke patients with near complete or complete recanalization post MT which may not address the current practice of using IAT in those patients with no or suboptimal recanalization post MT (rescue treatment).^1^ Therefore, an essential knowledge gap in use of IAT is not addressed by the current meta-analysis. The therapeutic benefit of IAT for rescue treatment cannot be ignored since an increase in mRS of 0–2 has been reported in observational studies in patients with no recanalization and those with partial recanalization after MT.^1^ Moreover, surveys of practitioners confirmed the equipoise regardless of recanalization status.^1^

Although the meta-analysis suggested a potential benefit of IAT in improving functional outcomes among patients post-MT, the potentially increased risk of sICH, inadequate sample sizes, and lack of data from Europe and United States is unlikely to result in change in new guidelines compared with the existing recommendations.^5^ A large definitive RCT is still required that provides generalizable information regarding risks and benefits of IAT with high certainty and more information in sub-groups among acute ischemic stroke patients undergoing MT.
